# The Legal Doctrine of "Fact" in Lunacy, and the Case of George Clark

**Published:** 1862-04

**Authors:** 


### Art. VII.—THE LEGAL DOCTRINE OF "FACT" IN LUNACY, AND THE CASE OF GEORGE CLARK.

On the 27th of February last, George Clark, a cabinet-maker, 45 years of age, was tried at Newcastle, before Mr. Justice Willes, for the wilful murder of Mark Frater, a tax-gatherer, on the 1st October, 1861. The prisoner pleaded "Not Guilty," and he refused to be aided by counsel, stating that he would defend himself. Upon this the Judge interposed, and said, "Have you any counsel?" *Prisoner.*—"I will address the judge: I will address the power." *Judge*—"Don't you think you had better let the court assign you counsel?" *Prisoner.*—"No, none." The Clerk of Arraigns was proceeding with his duty, when the prisoner interrupted him with the remark, "It is my duty for to address the court first." *Judge.*—"The jury will be in the box, and if you wish to object to any one of them, you may do so." *Prisoner.*—"I have something relating to the high honour in this town, and not only in this town, but in England. I may have to address the power." The jury was then sworn, and the trial proceeded.

The prisoner is described as presenting "but little appreciable difference in appearance" from that which he presented at the coroner's inquest, and when examined by the magistrates shortly after perpetrating the murder. "His face was rather pale, haggard, and sallow, as it was on the occasions referred to;" and although "that hasty irritability so conspicuously displayed at the inquest and at the magisterial investigation was evidently somewhat allayed, there was still an air of wildness in his aspect."* He frequently interrupted the counsel for the prosecution while stating his case with such remarks as "Hear, hear;" "Hold your tongue;" "It wont do that;" "Complete lies. Sit down, my good lad;" and he occasionally made use of still more offensive ob-

---

* We make use in this article of the graphic report of the trial published in *The Newcastle Chronicle and Northern Counties Advertiser*, March 1, 1862.

servations. At one time, indeed, it was necessary for the judge to interfere and caution the prisoner that he should be compelled to have him removed from the court unless he remained quiet.

From the evidence it appeared that, about half-past nine o'clock on the morning of the 1st October, 1861, while Mr. Frater was conversing with a friend on the pavement in front of his office door in Blackett-street, Clark came up to him from behind, and stabbed him in the neck with a sharp-pointed knife, inflicting a wound, from the effects of which Mr. Frater died in about ten minutes. The motive for the crime seemed to have arisen from Mr. Frater having distrained Clark's tools for unpaid dog-tax, in the month of May preceding. It was suggested in the case for the prosecution, that Clark would probably seek exemption from the consequences of his act on the ground of insanity; but so far as the prisoner's observations in the course of the trial assumed any consistency (for in the main they were irrelevant, inconsequent, and often incoherent), they were intended to convey the impression that Mr. Frater had killed himself voluntarily. This, indeed, was the defence (if defence it could be called) set up by the prisoner.

It is important to note, in the further consideration of the case, that the question of the insanity of the prisoner, that question upon which the subsequent great interest of the trial entirely depends, was brought prominently before both the judge and the jury at the very commencement of the case. "It was an unpleasant duty," the counsel for the prosecution, Mr. Davison, said, in addressing the jury, "to prosecute a man undefended by counsel; but he trusted he had not gone beyond what he ought to have done. It was, however, his bounden duty to call attention to what might appear to them a defence, and what might possibly be set up by the prisoner—a defence on the ground of insanity." Mr. Davison then briefly dwelt upon the law of the subject, and terminated his address, by saying that, "It might possibly be that the prisoner had some monomania on this subject, and he only asked them, on reasonable and satisfactory evidence, to come to a conclusion." Thus the attention of both the court and the jury was fully directed, at the very outset of the case, to the probable significance of the prisoner's own actions and language while before them, as well as to those portions of the evidence to be submitted to them, which might throw light upon his mental condition.

It is not necessary that we should dwell upon the evidence of the murder. The crime had been perpetrated in broad daylight, in a busy street, and in the presence of one person. The brief struggle consequent upon the murderous blow inflicted by the prisoner had been witnessed by two other persons, who secured the murderer and disarmed him while he still stood by the side of his victim. Moreover, the prisoner at the time of the murder, made no attempt to disavow the act, and he remarked to one of the persons who first laid hold of him, when the knife was taken from his hand, "It is all right; he robbed me, and now I have paid him." To another witness who helped to secure him, and who asked him what had induced him to stab Mr. Frater, he said that it was on account of the dog-tax; and when he was taken to the police-office he remarked to the officer in charge, "You may charge me with wilful murder."

We shall cite *verbatim* such portions of the evidence as will best enable us to judge of the mental condition of the prisoner prior to and at the time of the murder, as well as subsequent to that period, and when on his trial. After the first witness examined, Mr. T. S. Horn, had testified to the fact of the murder, while he was conversing with Mr. Frater, the judge, addressing the prisoner, said:—

Have you any questions to ask the witness?

*Prisoner.*—My noble power, gentlemen of the jury—

*The Judge.*—Your time has not come for that. That will be after all the witnesses have been examined. In the mean time, you have a right to ask the witnesses any question you think proper. Do you wish to ask this one any?

*Prisoner.*—Decidedly, my noble power. This gentleman here says he was coming along Northumberland-street with Mark Frater. I was standing at the corner of Pilgrim-street, and he came along towards Blackett-street. This gentleman stood talking to Mark Frater, with his left side towards the wall of his door. The opposite side was the north side, and he stated that he was standing with his left side towards mark Frater. I deny the words.

*The Judge.*—Were you standing at the left or right side of Mark Frater?

*Witness.*—I was standing at his right side—he upon my left side.

*Prisoner.*—My noble power, he was standing then as he was there (pointing); the door is yonder (pointing) going into Mark Frater's office—of course, face and face together—how came this man standing at Mark Frater's right side? You see here—the man is here, and Mark Frater was standing in this direction. Of course, he said he was standing at his right side—left side rather—but I say he was standing at the left side. I took the knife from Mark Frater. Mark Frater might be an antagonistic of people in England, and even in Newcastle intemperately insane, and we have often people in this town that often put an end to themselves by the intemperate insane system. We have men that represent themselves with pistols and blow their own brains out; but I reject the word, and I say they do not blow their own brains out, but I say they take the lives of theirselves by their intoxicating system. It might be the case again very likely, and a man might represent himself with a rope round his neck and he might hang himself. (The prisoner here became very incoherent in his remarks, and repeated what he had already said.) I say that Mark Frater had the knife in his possession when I was standing at the opposite side of Blackett-street, and that I went forth and took the knife from him, and whipped it down his back.

*The Judge.*—Had he any knife ?

*Witness.*—None, my lord.

*The Judge.*—Do you wish to ask any other questions of the witness ?

*Prisoner.*—All of them as they come on.

*The Judge.*—Do you wish to ask any other questions of this witness ?

*Prisoner.*—I say this witness is wrong in his explanations before the bar.

*The Judge.*—Do you wish to ask him any other questions ?

*Prisoner.*—None.

When the second witness, Mr. J. Dalrymple, who had been one of the first persons to seize upon him after the murder, had given his evidence, the prisoner said :—

I kept a dog. This witness took the knife from my hand as I took it from Mark Frater's hand. When Mark Frater took the knife from his sleeve and put it in his neck I went and put it down his throat. The knife was in Mark Frater's hand, and I took it from his neck. I took the knife from Mark Frater's hand, and this gentleman (witness) took the knife from my hand. (To witness)—Did you see me " represent" the knife from Mark Frater's hand ?

*Witness.*—No.

*Prisoner.*—Hear, hear. That is a point for Clark.

*The Judge.*—The last witness said he saw you thrust towards Frater's neck, but he did not see the knife.

*Prisoner.*—I took the knife from his hand as well as this gentleman took it from my hand.

*The Judge.*—He said he did not see it.

*Prisoner.*—A point for Clark. He is not guilty yet.

Upon the evidence of the third witness, Mr. R. Wilson, who had aided in securing him, the prisoner said, addressing the judge :—

My noble power, this gentleman here was not on the place when Mark Frater struggled with Clark, the prisoner. It was a few minutes after when this man made his appearance when this murder was committed by Mark Frater. There were plenty of gentlemen passing, but they would not come to prevent this murder coming to pass in the street in broad daylight. I say he was not on the place. It was a few minutes after when this man made his appearance.

*The Judge.*—That is what he says.

*Prisoner.*—Hear, hear. On these grounds, the prisoner Clark is not guilty of the murder.

*The Judge.*—Do you wish to ask any other questions ?

*Prisoner.*—Yes ; that is plenty for me.

The fourth witness for the prosecution, Mr. John McGilvray, on being sworn, said :—

I am a draper in this town, and live in Leazes-terrace. On Tuesday, the 1st of October last, I was crossing Blackett-street in the direction of Northumberland-street, at half-past nine o'clock in the morning. I know Mr. Frater's office, and I observed a struggle going on between two men; one was Mark Frater, and the other the prisoner at the bar. I could not say that the struggle was between them alone.

*Prisoner.*—Hear, hear.

The prisoner held a knife in his right hand, and Mr. Horn had hold of his right hand, and I seized hold of his left. The knife prisoner had in his right hand was half covered with blood. Mr. Frater was turning to go into his office. He had his hand to his neck, and I observed blood coming over his hands as he was going up the steps. I could not say there was any blood on the prisoner's hand.

*Prisoner.*—Hear, hear.

*Witness.*—Immediately after Mr. Rayne drove up and attended Mr. Frater, and I went with the prisoner to the Prudhoe-street Police-station. That is the knife [produced]. Prisoner said that Mr. Frater had robbed him, and he had paid him now.

*Prisoner.*—These are different statements, you must remember. Points these for the prisoner Clark.

*Witness.*—He said a good deal, but I do not remember any other statements. He talked about taxes a good deal, and about paying them to the Queen, and not to any other person. *He seemed to talk as if there were a fictitious Queen, and they did not pay taxes to the real one.*

*Prisoner.*—I did not say the Queens. I said the Queen was not receiving the dog-taxes, but the government was receiving them. But that will come on after.

*Witness.*—He said he hoped Mr. Frater was dead.

*Prisoner.*—My noble power, this gentleman was not there at the time this murder was committed with Mark Frater and done by him. He is a stranger to me, but it is my duty to know my position as well as others to come forth and receive Clark as prisoner. This gentleman is a stranger, but he may come forward against Clark the prisoner. He might come forth in amongst the crowd, but I can state for myself that he was never there when this took place between Mark Frater and Clark the prisoner. My noble power, on these grounds I say Clark, the prisoner, is not guilty before the bar at the crown, or rather the crown at the bar.

*The Judge* (to witness).—You went up as the struggle was going on?

*Witness.*—Yes.

*The Judge*—Was Wilson there when you got up?

*Witness.*—No, sir.

*The Judge.*—Was Dalrymple?

*Witness.*—No, sir. Horn was.

*Prisoner.*—Now he says a second one. The other said he took hold of my hand. I have only two hands; and this took hold of one, where was the third to come from? That may be a mistake.

*The Judge.*—That does not seem to come to much, because two persons might lay hold of one of your arms.

*Prisoner.*—They said they took my hand. I say this man was not there when Clark, the prisoner, was with Mark Frater. It is my duty to watch the points, as well as yours to watch the movements.

On the evidence of a subsequent witness, a policeman, who had seen Clark near Blackett-street a few minutes before the murder was committed, and to whom the prisoner had spoken as he passed, the prisoner said, addressing the judge :—

*Prisoner.*—I did see this gentleman policeman, my noble power, pass me and go on Blackett-street, about that time in the morning. I did not go to work that morning, as I had got a little wee drop of drink, and I thought a little walk would set me right for work after dinner. It was not much, only a glass of ale. On these grounds that this gentleman went past and never said anything to me, I said nothing to him. I was not intoxicated with drink, except I had taken a small glass of ale. It was not a gill glass of ale; only a small one. But as I am only a young man I take my pleasures and then go to work to keep myself honest from all temptations and to live quietly and soberly in the world. On these grounds I say Mark Frater came and stabbed himself. Mark Frater came and put the knife to his own throat.

*The Judge* (to witness).—Did you speak to him ?

*Witness.*—He spoke to me, my lord. He said it was a fine morning, and I said it was.

*Prisoner.*—I believe you are telling a lie.

Mr. Septimus William Rayne, the medical officer of the police force of Newcastle, was next examined. He was passing through Blackett-street at the moment when the murder was committed, and gave such aid as he could to Mr. Frater. Having testified as to the nature and character of the wound which had been inflicted, and the cause of death, he deposed further as follows :—

*Mr. Davison.*—Could you say whether Mr. Frater could have inflicted that blow on his own neck ?

*Witness.*—No, he could not have done it in that place.

*Mr. Davison.*—I believe you have seen the prisoner since the encounter ?—Yes.

*Mr. Davison.*—On more than one occasion ?—I have.

*Mr. Davison.*—Have you formed an opinion as to the state of the prisoner's mind ?—Do you mean at the time it took place ?

*Mr. Davison.*—At that time. The time when this was done.

*Witness.*—Yes. I think he was a man of strong passion; he was an eccentric man. These opinions are formed from conversations I had with him immediately after he was with Mr. Frater. I am of opinion that he was not acting under delusion at the time he killed Mr. Frater.

*Mr. Davison.*—Is that your opinion now as to his state of mind then ?—I have not altered my opinion with regard to his state of mind at the time.

*Mr. Davison.*—Have you formed any opinion as to the present state of his mind ?—The last time I visited him I found him a religious monomaniac, but not before. It would be last Monday when I saw him last. *He talked in that way that he was Christ; that there was a power above him; and that he had been in Palestine.*

*Prisoner.*—That is a thing we have nothing to do with here.

*The Judge* (to witness).—He said what?

*Witness.—He said there were two Christs, one was Jesus Christ, and he himself was Christ.*

*The Judge.*—You mentioned something more which you called religious monomania, which I should call religious mania if he was sincere in what he meant.

*Witness.—He said he was* 45 *years of age, and that* 60 *years ago he dwelt in Palestine, and that he was sent here as a supreme power.*

*Mr. Davison.*—Is there any other conversation you now remember?

*Witness.*—No: I believe it was superstition to a great extent.

*Mr. Davison.*—You believe that the state of mind he was in on the 1st October was such as, when he struck Mr. Frater, to enable him to have a knowledge of what he was doing?

*Witness.*—I am of opinion that he went to murder Mr. Frater; that he had preconceived it; and that he knew what he was going to do.

*The Judge.*—Was this what you learned from conversations with himself?

*Witness.*—From himself. I have had frequent conversations with him.

*Prisoner.*—My noble power, I am happy to hear Dr. Rayne state his noble opinion. He states before the crown at the bar that he was ten minutes after the murder was committed. I believe it was just thereabout. The point is, he did not see Clark "represent" the knife to Frater. On these grounds he is not a witness to convict me to be a prisoner to the court.

*The Judge.*—I understood Dr. Rayne to say that death took place within ten minutes at the outside.

*Mr. Rayne.*—From the time I saw him on the steps.

*Prisoner.*—That is what he says. All the work was done when he appeared in his carriage. After that it was finished. This witness (Mr. McGilvray) had Clark in hand when he (Mr. Rayne) came forth. On these grounds then there is no ground. He said again that he put his fingers in the 'oond. If this gentleman had been a medical skilful gentleman it was his duty to bind up the 'oond, not to put his fingers into the 'oond to destroy that life he could not prevent or give. My noble power, we have had in this town one murder taken place. It is the duty of the medical gentleman to bind up the 'oond as quick as he can that he might not vanish his life in distress. But he represented his hand now to cause death to appear. When he put his fingers into the 'oond was he not causing murder to be committed? I say that he was one of the instigators to the late Mark Frater. I say, on these grounds, he was one of the instigators to cause death to appear in the late Mark Frater. I say did he see whether he was intoxicated or whether he was excited in mind? As far as I can learn from those witnesses, there is no opinion as to the state in which Mark Frater was in, or whether he was in his right wits on this morning. He may have been agitated when he took the knife from his sleeve and put it in his own neck, and which the prisoner Clark went and put down his throat. I never put the knife in his neck; no, not at all. On these grounds there is no evidence to commit Clark; no, not at all.

*The Judge* (to Mr. Rayne).—Do I understand you to say that from recent conversations you have had with him you are of opinion that his mind is now in a diseased state?

*Witness.*—Yes, my lord.

*The Judge.*—Have you had opportunities of observing persons whose minds were in a diseased state?—I have.

*The Judge.*—You attribute the wanderings and strange statements to which you refer to disease of the mind?—I do.

*The Judge.*—You are of opinion, therefore, I presume, that those wanderings were not feigned wanderings?—No; I have never broke him down in his religious wanderings at all.

*The Judge.*—Have you had the opportunity of observing his conduct to-day?

*Witness.—Yes; it is somewhat similar to his conduct throughout the whole proceedings. He exhibited the same conduct before the coroner and the same before the magistrates.*

*The Judge.*—Have you seen him in prison?—I have, three or four times.

*The Judge.*—And has his manner been consistent with the manner he has exhibited to-day?—Yes, in many respects. He never showed any religious mania until last Monday.

*The Judge.*—I will not speak of that monomania you alluded to, so many errors have arisen in connexion with monomania. Do you think these notions he has represented were not feigned statements, but were representations of what was going on in his own mind?

*Witness.*—I have not the slightest doubt of it.

*The Judge.*—According to your judgment, then, he is an insane person?—He is at the present time. I have no doubt of that.

*The Judge.*—I understand you to say that his recent state is consistent with his not understanding what he was doing on the 1st of October?

*Witness.*—I have not the slightest doubt that he knew what he was doing on the 1st of October.

*The Judge.*—That is the result of your examination immediately after the occurrence?—It is.

*The Judge.*—From this recent knowledge, can you form any judgment as to what was his state of mind on the 1st October?

*Witness.*—I think on the 1st of October he was an eccentric man, with a revengeful spirit, but he perfectly knew what he was doing. [Prisoner: Hear, hear.] I think he was a religious man, and through the instructions of the chaplain he has taken a controverted view of it.

The police-officer in charge of the Prudhoe-street Police-station, on the morning of the murder, deposed that, when the prisoner was brought to the station, and charged by him with the murder, he remarked, "Oh decidedly, oh decidedly, he robbed me and I've robbed him;" and further, that while he was being searched the prisoner said, "You may charge me with wilful murder."

James McDonald, a fellow-workman of the prisoner's, deposed to knowing him for fifteen years, and to having heard him complain in the workshop of the excessive distraint which had been made upon him for the dog-tax. He also stated that he had heard Clark say many times that Mr. Frater had murdered him, and that he would murder Mr. Frater. "We," said the witness, speaking of himself and fellow-workmen, "took that as nonsense." Further, this witness on being asked by the judge.—"Have you had any opportunity of seeing whether he was sane?" replied, "I have seen him from time to time—a great many times." *Judge.*—"Did he conduct himself like other working men?" *Witness.*—"*No, sir, he did not; he has always been a sort of excitable, curious man.*" *Judge.*—"But what has he ever done that you know that makes you say so?" *Witness.*—"Well, I have been where the men told me he had taken up a chisel to strike them." *Judge.*—"Have you ever seen him do anything strange, or out of the way?" *Witness.*—"No, nothing, except when he struck them." *Judge.*—"Have you heard his talk here to-day?" *Witness.*—"Yes." *Judge.*—"Has he conducted himself in the same sort of way as he has done to-day during the time you have known him?" *Witness.*—"*Oh, yes, the time I have known him talking the same as I have known him to-day.*"

Wm. Cook, a bailiff's follower, deposed as follows:—

I, with Francis Bone, on the 18th May, distrained upon some of the prisoner's goods. He lived then in St. Nicholas' Churchyard. Mr. Frater was collector of land and assessed taxes, and we were employed by him. It was for six shillings unpaid dog-tax. We took a saw and a cramp, and removed them to the Black Bull public-house. I saw the prisoner soon after the 18th May, and had some conversation with him. That was on a Monday, and I met him near St. Nicholas' Church. He asked me where the goods were, and I told him. He said Mr. Frater had better return them, or it would be worse for him. That is what he said, to the best of my knowledge. The goods were taken from the Black Bull to the office of Mr. Frater. Perhaps four or five days after the first conversation, he met me again in the Bigg Market. He asked where the goods were. I said they were at Mr. Frater's office, and that if he would go there he would get them. He said that if Mr. Frater did not return the goods to him it would be worse for him. *Perhaps four months before the 1st October I was coming out of Mr. Caldwell's, draper, and Clark met me in the face coming out with a small parcel in my hand. He asked me about his goods; and he said I might tell Mr. Frater, "I have a mission to perform between God and myself, and if the goods are not returned he shall pay dearly for it."**

* This fact was also stated by Cook at the coroner's inquest, but with an important variation in the phraseology. He then said:—"On the Tuesday, the morning of the making of the distress, Clark stopped him at St. Nicholas' Churchyard-corner, and asked him where the goods were. Witness replied, 'At the Black Bull.' Clark said if Mr. Frater did not return them he would make him pay dearly for it. He told Mr. Frater what he said. Mr. Frater replied, 'The poor silly booby.' He met Clark about ten days after that in the Bigg market, and he asked where

*The Judge.*—Have you anything to ask him?

*Prisoner.*—My noble power, he is an old wife, and should have a good memory. I never spoke anything to this witness but once. I asked Mr. Cook where my tools had gone to, and he told me that Mark Frater had them. I said it was a very hard case that I should lose my tools of the value of 25*s.* to pay 6*s.* of debt, I myself working for my living honestly and uprightly. And I said it was not right for a thief like Mark Frater to come into my property without a legal writ from the magistrates, and take possession of my property that I had in my estate. When he represents these gentlemen (the bailiffs) he comes forth with nothing in his hand, but they came on their own bottom, and took my tools, value 25*s.* to pay 6*s.* Is it not most disgraceful that Mark Frater should take 25*s.* worth of tools to pay a 6*s.* debt? Is there any gentleman that acts honestly between man and man that would take and impoverish the working classes like that. Suppose Mark Frater took the value of them, there was the costs upon them, my noble crown. What did he do with the other money? Was he going to pocket it? He must have been a scoundrel and a knave to take them because the dog-tax was not paid. I never refused to pay this amount of money. I only refused Mark Frater's clerk at six months' end. I told Mark Frater's clerk that I would pay him at the twelve months' end, and pay 12*s.* instead of 6*s.* He being a blackguard and a reprobate would not wait, but sent those men to take and value my property. I leave it to my noble friend that they had no legal right from the magistrates of this town to do so. They took upon themselves to do this work; *and if your noble power will take upon you to let this work go on, you will find more work in England than there has been for years past.* I hope my noble crown at the bar will look out a case, and will not allow those individuals to rob and plunder the working classes of their own.

The last witness examined on the part of the prosecution was the chaplain of the gaol, the Rev. Robert Shepherd, jun., and his evidence is reported thus:—

*The Judge* (to Mr. Davison).—I want to know whether he (the Chaplain) has observed anything about the conduct of the prisoner. Perhaps you will ask the questions.

*Mr. Davison.*—You're chaplain of the gaol?—Yes.

*Mr. Davison.*—And have been so during the whole time that the prisoner has been in custody?—Yes.

*Mr. Davison.*—As chaplain of the gaol, have you frequently seen him during that period up to the present time?—Yes; nearly daily.

*Mr. Davison.*—Have you observed anything about his conduct upon those occasions?—Yes.

*Mr. Davison.*—What have you observed about him?

*Witness.*—I observed a very peculiar manner about him. The first thing that I may state was whilst I was in one of the rooms or cells along

---

the goods were, and he said in Mr. Frater's office. Clark then replied that *he had a mission to perform between God and himself, and if the goods were not returned he would perform it very shortly.*"—*Times,* Oct. 3.

with some other prisoners. I had some conversation with the prisoners in that cell, and he said, "Are you going away without praying with us?" I said, "Do you wish me to pray with you?" He said, "Yes."

*Mr. Davison.*—When was that?

*Witness.*—About the first or second day that I saw him in prison. "Very well," I said, "I will kneel down and pray with you." I did so, and as soon as I was done he commenced singing. Of course I got up, and immediately went away with the officer who accompanied me to the cell.

*Mr. Davison.*—I don't know that that's quite clear. As I understand you, he sang on his knees?

*Witness.*—Yes; he commenced to sing on his knees.

*Mr. Davison.*—What was he singing?

*Witness.*—Would my lord wish me to describe the matter?

*The Judge.*—Was it secular music or sacred music?

*Witness.*—Nothing but a garbled kind of matter. A sort of singing that I could not understand.

*The Judge.*—What was the tune?

*Witness.*—It was some kind of humming.

*The Judge.*—Are you musical?—A little.

*The Judge.*—You know what I mean by asking the question?—Yes.

*Mr. Davison.*—Well, now, what next can you remember that you observed as peculiar?

*Witness.*—Soon after that I saw a gentleman of the name of Gibson, and he told me that Clark had been in the habit, before this matter took place——

*Mr. Davison.*—Well, Mr. Gibson told you something, in consequence of that?

*Witness.*—He told me Clark *had been in the habit of taking a pint of olive oil every week. Soon after that, I saw Clark, and asked him the reason why he took this pint of olive oil every week? He said he did it according to a verse he found in the Bible, that a man must keep his lamp well trimmed and burning bright to show him to another world.*

*Mr. Davison.*—Now, can you say how long after he came under your notice that that took place?

*Witness.*—Probably a week or a fortnight, or soon after.

*Mr. Davison.*—Was there any other matter that took place at that time that you can mention in giving evidence to the jury?

*Witness.*—Nothing, *but his general conversation on matters, which led me to the conclusion a few days after I had been with him that he was insane, and I reported that in my journal.*

*Mr. Davison.*—Any other fact or conversation of which you can give the details now?

*Witness.*—Well, in conversation he has told me to go out, and that I had no business there. He has said I am So-and-so, and have a mansion in another world. Once he said, "What would you think if I was the Christ Himself?"

*Mr. Davison.*—Any other speech or observation?

*Witness.*—Well, from those peculiarities which I observed in his conduct, I was naturally led to make inquiries of those who had known him for years past.

*Mr. Davison.*—Is there any more conversation between you, remark or speech that he has made, or any other act that he has done?

*Witness.*—Yes; he told me that this world—the globe on which we live—is no part of the great world; there is another world beyond, and the Great Spirit who belongs to the other world has nothing to do with this world; that this world rests upon air; the other, sun, and moon, rest upon a cherubim.

*Mr. Davison.*—Is that all you remember?

*Witness.*—I did not expect to be called on, or else I might have been better prepared.

*Mr. Davison.*—Is there any act or deed during the time you were with him, or rather on the occasion you saw him, indicating a peculiar nature, or was it only the conversation you had with him?

*Witness.*—His conversation and manner, ordering me out, and saying, " You're a fool."

*The Judge.*—Was that when you spoke to him on religious subjects?

*Witness.*—No; it was more in reference to general conversation about this act which he is alleged to have committed—the murder of Mark Frater. *He said he rejoiced in the act, and would do it again.*

*The Judge.*—Tell all you recollect, because it is very material. Recollect yourself as well as you can, and give us the whole of what passed between you.

*Witness.*—On one occasion, when talking about the murder of Mark Frater, there was some noise had taken place in another cell immediately below the one in which he was placed. Clark said they were a noisy, dirty class of men, and if he were permitted to go down he'd kill every one of them.

*The Judge* (to prisoner).—Do you wish to ask any question?

*Prisoner.*—Yes, my lord. We have had a vast of old wives this morning in the Court. We have had a vast of old wives, and I think that's one among the rest. He's come here with nothing particular, I think. I think he must be a fishwife, or something of that sort. He would never pass for a gentleman of any principle to come in such a state before the Crown as he has done, and delivered such evidence not worth hearing. I leave this to your Honour to take into consideration. I leave this to my noble Crown and the bar to decide this matter. I think it is not worth me speaking about it.

The judge now asked the prisoner if he wished to say anything to the jury. The prisoner, addressing his Lordship, replied: " If you've done, I will commence with the jury;" and, after a pause, he said:—

My noble power, gentlemen in the jury, I have paid dog-tax money to the Corporation, but in the year of 1860, I left my workshop and went to London. Going to London, I inquired there at one of my friends if they could give me any good intelligence where I could get a first-rate counsellor, which they did. After I seed this honoured gentleman in London, I addressed myself before the gentleman, and I stated that I had a letter. I represented this letter before the gentleman, and I asked this gentleman if he would back this letter. He looked at it, and after he read the letter, he said unto me "My dear sir, I cannot back this letter under 5*l.* 10s. Agreed to. The incoming morning, I received a letter from the Lady the Queen in England. This letter, then, contained—"My dear friend, Mr. Clark, I received a letter from So-and-so in the evening, where I am very happy to state in my deliverage in the letter that I do not take any taxations from the lands whatever;" that the Lady the Queen stated in the deliverage in her letter, stating that she never received any taxation—regarding taxation of dogs, but the Corporations of each town received this taxation to pay in the national bank, to keep their salaries moving. Again, my noble power, gentlemen in the jury, the lady the Queen stated—"My good man, Mr. Clark, I receive nothing in England but duty rates, from port to port, and from river to river." On these grounds, then, the lady stated in the letter that it was passed over to the under government, that they had to receive all taxations regarding dogs throughout the land to keep up their national debts. The Lady wrote in the letter, and stated that she received no borough rates whatever from this land or any part of the globe; that she received no income-tax whatever. All she received was the kitchen dues; but if this kitchen hadn't a cellar, or a cellar beneath a certain depthness of earth, the Lady the Queen of England did not receive any dues from that house whatever; but the Corporation of each town received dog-taxes, and they passed the bill on to the under Government. That the lady the Queen wrote in her letter that she never signed the bill to pass this dog-tax throughout the land. On these grounds, then, it is a false statement given to the public, but then the under Government takes in their possession to rob and plunder the working classes of this dreadful thing. That the Lady in England stated in her letter that she did not handle no taxatiou whatever in the lands. All she received was the river dues, and the duties from port to port, while they are in these letters. Again, my noble power, gentlemen in the jury, the Lady wrote in the letter, "My dear friend, Mr. Clark, as you represented the whole before me, I give you full particulars, that all banks that you have in England is not signed by me, but is signed by the under Government of this town; that if a bank breaks, then this bank is not enrolled by the Lady Crown—the Lady the Queen in England." On these grounds, then, if the Corporation of this town is in poverty, is in distress, then the branch bank did break, that they could plunder from these banks to help up other distressed boroughs in England. That the Lady of England did not sign the bill. Therefore, I consider, then, my noble power, I think it is only a rogue from London to drive a thief out of Newcastle, because he'll not pay this dog-tax money to keep up their salaries; but for all that's passed yet, not one single man can bring Clark before the hammer, and say that he is guilty of this dreadful murder. Gentlemen in the jury, this is the fact, I received from the Lady the Queen herself.

*The Judge.*—Do you wish to say anything more?

*The Prisoner.*—Yes, my noble power. Gentlemen in the jury, when we showed that knife by the witnesses, they said they found that this knife had been bent. Supposing, then, that this knife had been bent, it must have been bent in the presence of Mark Frater. The witnesses say that they heard chopping from this knife ; but I say Mark Frater was a complete impostor against the working classes in general, and he was one of those instigators if a person was in arrear of rent he took possession of the property, and very likely this knife might be one of those things that he took in possession. When a man is hard up in the world, perhaps he might get that knife from a shoemaker, and perhaps he has taken the liberty of grinding the corner off that knife to do his own work, and be an honest, upright man, rather take charge of his own property, as have property he could not pay. Supposing then, my noble Crown and gentlemen in the jury, that this had been the proper knife that had done this trick to Mark Frater, would you imagine for a moment this knife could be bent by any wound struck from the neck ? Is there any bones in the neck ? There may be some cross bones, perhaps, to bend this knife down, but it seems to me that it is a strong-cased knife, and I think by the appearance of the knife, it must have been bent before it entered into his neck. I have not the least doubt but it might do so. A man that is instigated even destroys his own property by the guilty way he is going on. Again, my noble power, gentlemen in the jury, we'll take the knife in a different light. Again, supposing this knife had been only a breakfast knife instead of a strong-cased knife like that, do you imagine, my noble power, if the knife be straight cut to the bone, that knife is such to show that it is the proper knife that's done the murder ? It is a mistake, but it's like all things past against the working man; but perhaps the working man may prove to have better brains than the old wives that have come before you to-day. Again, supposing, then, that if this knife had been run into the man's side, where the ribs and bones do dwell, perhaps by the pressure of that it may have bent the blade, and give something like the same knife that done the murder. Now, take the thing in clear light before you. My noble power, supposing then that it had been among the ribs and in some other part in the bones of the body, it might be bent as the pressure under bent the blade, and given this to be the right knife that's done the murder. But on the contrary, as I said before, going into the neck, where there is no bone, but flesh and veins, I say it is not the knife that's represented before the Crown at the bar, and if there is any one can contradict it, I thank him for doing so. I am done.

His Lordship then summed up as follows :—

Gentlemen of the Jury,—The prisoner, George Clark, is indicted for the murder of Mark Frater upon the morning of the 1st of October last, and there will be two questions to which you will have to direct your attention. The first is, whether you are satisfied that the prisoner was the man who inflicted upon Frater the wound which caused his death. If so, it will be your duty to convict him of the murder, unless you are satisfied with respect to the second question. That is, was the prisoner in such a state from disease—not from exaggerated passion—from disease of mind, that either he did not know what he was doing, or that he did not know what he was doing was wrong. Whether in your judgment the prisoner knew right from wrong, is about the clearest, as well as the shortest, way of putting it—whether he was insane, or whether he was insane to such an extent as, having regard to the law as I shall lay it down, would excuse him from the crime, and subject him to be locked up for the remainder of his life as a madman, instead of being punished as a murderer. Of course, it is hardly necessary to observe that persons are punished by the law for crimes, in consequence, as much of the intention of the mind, as of the act which the mind instigates the person to do. There must be a guilty mind to make out the offence, and a guilty mind is not consistent with a state in which the man does not know right from wrong. In other words, whilst the public must be protected—where life and property must be protected by punishing all persons who commit acts which they know to be wrong; on the other hand, it would be folly—almost blasphemy—to punish a man for an offence to which he has been instigated, not by his own guilty will, but by an affliction sent upon him by Providence itself. You would not punish a madman. However, we must take very good care that when a question of this kind is brought before the jury, they are thoroughly satisfied that the man was not only mad, but that his insanity was of such a character and to such an extent that he was incapable of knowing what he was doing, or knowing he was doing what was wrong. The law upon the subject has been laid down in comparatively recent times by the judges' opinions, to which the learned counsel for the prosecution referred in his opening. In consequence of a well-known case, the House of Lords put questions to the judges, and the judges answered them in this way. There is no doubt that this is the law, and that it is the duty of any one sitting upon a question of this kind to lay down the law. I will tell you what that law is. It is not like the question that arises whether a man is capable of making his will. If a man is shown to have a delusion upon any subject, no matter how remote it may be from the question whether he can form the intention expressed by his will or not—if he is shown to have a morbid delusion in his mind, he is not allowed by the law to make his will. Take the instance of a man who fancies that his head is made of glass—not a very uncommon delusion. A man supposes his head is made of glass, and though, as capable of attending to his business, he is capable of performing an intention—of knowing what he is about, of knowing what he is doing, and knowing whether what he is doing is right or wrong—such a delusion as that makes him incapable of making a will, because he is not of sound mind. But that certainty would not absolve him from answering for any crime which he might commit. If a man is a stark madman, if he is a person whose mind receives from his senses erroneous impressions—especially when he was striking a man, he might suppose it was a dog or other animal—of course you would deal with that case simply and at once. You can form your own judgment whether this is that case, or whether the prisoner is capable of receiving correct impressions of what is going on before him through his senses. *In one respect, it is an advantage that the prisoner declined to be defended by counsel, and you had an opportunity of observing for a long time his conduct at, of course, a very critical*

*moment of his life.* But there is the other case, where a man is labouring under a sort of partial delusion to which I have referred. In that case, the law is this—that although he is labouring under a morbid delusion, yet such delusion will not excuse him, unless it were true that it would, in point of fact, form a justification for the act being done by a sane man. If the delusion is that a person is about to attack him and take his life, or rob him by force, or commit a burglary in his house, but only a morbid and insane delusion, that man is committing an act of that kind which would justify a person who was sane in killing that man. If that was the sort of delusion that existed in the mind of the person charged, then upon that being proved, the law gives him the benefit of taking it for granted that his delusion is true, and entitles him to be acquitted on the ground of insanity, because, if he were sane, it would have justified the act which he did. But if the delusion which a man has is that another has committed some great injury, has broken the law in respect of him, by depriving him of his property, or by slandering him, or by committing any other act of that description which would be an injury, but which would not, in point of law, justify the reeking vengeance upon the wrong-doer by an act calculated to produce death, then, notwithstanding such delusion, the law says it is no excuse for the offence which was done, and does not justify the jury in acquitting the prisoner upon the ground of insanity, because it would constitute no justification to a sane man if it were true. In point of fact, the law does so, because it acts upon people's fears, and it endeavours to protect persons from the murderous attacks of others by acting upon the terrors of those who may feel disposed to do such acts; and if a person has a particular delusion, but still has the power of knowing what he is doing, and that what he is doing is wrong, the law will make such a person responsible. Now, it was stated with respect to the mode in which this should be left to the jury, that it is this—that the burthen of making out a defence of this kind rests upon the prisoner or those who defend him; that the man is to be taken *prima facie* to be sane and to have sufficient reason to judge of what he is doing, and of the guilty character of what he is doing; and that assumption can only be removed by its being made out "clearly"—that is the word of the opinion—its being made out clearly to the satisfaction of the jury, either that he did not know what sort of act he was doing—the expression is, the nature and quality of the act he was doing, but that is, only a roundabout term for the sort of act he was doing—or that the thing that he was doing was wrong. That is the sum and substance of the law upon the matter. If you come to the conclusion, upon the facts, that the requisites of that law for an acquittal upon the ground of insanity are made out to your satisfaction, then you ought to acquit the prisoner on the ground of insanity. But you ought not to do that unless the evidence thoroughly satisfies you that the prisoner comes within the description of man I have laid down. Well, now, I have abstained from making any remarks upon the first question, because I really think I should be tampering with the facts if I did so, and shall, therefore, principally direct your attention to the other question. Now, first of all, you have this made out, that in the previous part of the year, the deceased man, Frater, who was a collector of the taxes, had distrained upon the prisoner for the dog-tax. The prisoner, according to his statement, had resented the act, and had spoken to other persons about it, before the morning of Frater's death; and he did on that morning state in the presence of at least two of the witnesses who have been called, that it was the dog-tax which caused Frater's death, and which led to it. Now, you will have to judge whether this was done under the passion of revenge, for Mr. Frater having made the distress, or whether it was in consequence of such an insane illusion or delusion as I have described. Now, I bring you at once to the scene of the death of Frater. Here his Lordship read over the evidence of the principal witnesses, and said, it is very material to see what the prisoner said about the time of this transaction. He said, "It's all right; he's robbed me, and now I have paid him." Referring to the word "monomania" used by Mr. Rayne, his Lordship said he had heard of a man being called "a homicidal monomaniac," having a sort of irresistible impulse to do an act of this kind, but that certainly had no place in the law. There is, he said, no such known to the law as a man being irresponsible because he has an irresistible impulse to kill another. If a man yields to an impulse of that kind, he is answerable by law, and I think, to say that men are afflicted by homicidal monomania, is to say that which has no weight in these cases, if they are to be determined according to the law of the land. As men of experience, you have had an opportunity of observing the manner of the prisoner here to-day, and you will form your own judgment, saying how far you think the sort of conduct he has manifested savours only of folly, or of ignorance, or of passion, or how far it may go to confirm the case that has been set up for the purpose of getting off. I do not say it in any invidious sense or at all as adverse to the prisoner, but as the shortest way of expressing it—for the purpose of inducing you to acquit him on the ground of insanity. Now, that is the whole matter. First of all, you must consider for yourselves whether it is proved that the prisoner did take away the life of Frater. If so, you ought to convict him of murder in itself; but if you are not satisfied, which will be the next matter for consideration, you ought to acquit him. If you answer the first question in the affirmative, the question whether he did take away the life of Frater, you will then have to consider the second question—whether in your judgment it is made out clearly to your satisfaction that the prisoner at the time he did the act was, by a disease of the mind, incapable of knowing what he was doing, or of knowing that he was doing wrong. You are the sole judges of this matter. You will have to consider whether you acquit him altogether, in which case you will say so by your verdict; whether you find him guilty of murder, being satisfied as to his sanity, in which case it will be your duty to say so by your verdict; or whether you are satisfied he was insane and to the extent and in the manner I have mentioned, which would amount to an excuse in point of law, on that ground, and in such case you would acquit him on the ground of insanity. These are all the observations I propose to make, and I must now leave the matter in your hands.

A verdict of GUILTY was returned by the jury, whereupon the prisoner exclaimed—"I plead not guilty. As the jury brought in a verdict of guilty, let it be so. I will take it."

The judge then proceeded to pass sentence in the following words:—

"George Clark, you have been convicted of the crime of wilful murder. You have been convicted of causing the death of Mark Frater, by stabbing him in the neck with a knife, and the evidence left no doubt that you committed that offence in consequence of preconceived vengeance, that you determined in your mind to revenge yourself upon the tax collector for the performance of his duty in having distrained upon your goods for the tax you were bound to pay to Government. You appear for a considerable time to have deliberated full revenge against him, and you appear days before to have been engaged in preparing weapons to bring him to an untimely end. No one could doubt on the evidence that you caused his death. And the only real question for the consideration of the jury was, whether you were in a state of mind to be responsible for your actions. *The evidence has satisfied the minds of the jury, as it has satisfied my mind, that you were in such a state of mind.* The jury, after lengthened consideration, have compelled themselves to pronounce a verdict of guilty; and that makes it my duty to pass upon you the sentence of the law. There were many circumstances in the case calling for the gravest consideration. Such were the circumstances mentioned by the surgeon and by the chaplain, with whom you had conversations, and which raised impressions in their minds which they have stated in evidence. *If I had entertained the slightest doubt on the evidence that you were not in a condition to understand perfectly the whole of what you were doing in depriving yourself of counsel and defending yourself, I should in the first instance have postponed the trial, and I should have postponed passing on you the sentence of the law. But I am convinced that you are a person of eccentric conduct and violent character—your character is eccentric beyond that of other men. No doubt you knew you were committing murder when you took Frater's life.* I must therefore proceed to pass sentence at once, hoping that it is possible, after those declarations you have made—after the determination to take away life—*after that dreadful state of mind you have been in since—rejoicing in the crime you have committed—rejoicing in shedding the blood of a fellow creature*—I do hope that you have sincere feelings of repentance to God, and that you will devote yourself to asking the forgiveness of the Almighty for the sake of Him who died for us all. You will have the assistance of a clergyman, and I do beg that you will take advantage of it. The sentence of the court is, that you be taken from hence to a place of public execution, and there be hanged by the neck until your body be dead, and that your body be buried within the precincts of the gaol. And may the Lord have mercy on your soul."

The following description of the prisoner's conduct during the trial, quoted from the report of which we have made use, forms a necessary pendant to the case :—

"The prisoner, on being placed in the dock, seemed in no way altered, either in looks or carriage. Had he been a martyr on his way to the stake, he could not have displayed greater firmness, or have gloried more in the work he had done. Throughout the proceedings, he was either unconscious of the terrible position in which he stood, or his assurance, considering the incontrovertible character of the evidence against him, amounted to recklessness. He conducted his own defence, cross-examined the witnesses, abused the counsel for the prosecution, argued matters of law with the judge, and bore himself bravely to the end. The man must be either a consummate impostor or mad. His language was flighty and incoherent in the extreme, and he was evidently unaware of the significance of many of the words he made use of. The tinny and slightly nasal tones of his voice, together with the conformation of his head, were symptomatic of a man of weak, if not erratic, intellect. There was not only not sense in his matter, but positive indications of mania of a very active form ; while his manner, gestures, and general braggadocio throughout the painful trial were no less significant of mental hallucination. His memory was good, since he could remember the details of his previous examinations, and he appeared to possess much of that ingenuity which many persons of weak intellect are known to possess. He was consistent in nothing but inconsistency. There was a thorough and pervading irrelevance and incoherence in every statement he made. His highest oratorical displays were never more than a tissue of words without meaning, accompanied by attitudes and gestures of the most extravagant character. He heard the verdict without emotion, and without change of colour. All that he deigned to say in reply to the judge, why sentence of death should not be passed upon him, was, that he pleaded not guilty ; but that as they (the jury) had given their judgment, 'so let it be.'"

The subsequent history of this extraordinary case is quickly told, and is not less extraordinary than the case itself. The good people of Newcastle, shocked with a sentence which would consign a man to the gallows whose eccentricity, if he were merely eccentric as the learned judge held, had, at least at the time of his trial and since the murder, differed so little from lunacy in appearance, that the difference could not be detected by ordinary understandings, took vigorous steps to obtain a respite of the sentence. Moreover, in the House of Commons, on the evening of the 17th ult., the Home Secretary, in answer to a question from Sir H. Willoughby, whether Clark had been respited, and whether there was any reason for supposing that the unfortunate man was insane, stated that the sentence had not been wholly commuted, but that the execution of it had been respited wholly on the grounds of the alleged insanity of the prisoner. "*Immediately after the trial,*" he said, "*the judge wrote to me to say*

*that he felt it his duty to take the first opportunity of bringing under my attention the peculiar circumstances of the case. He agreed with the verdict of the jury, because there was no evidence that, at the time of the commission of the crime, the prisoner was insane; but his conduct at the trial was of the most eccentric and extraordinary character; and it was the opinion of the medical officer of the prison, who was stated to be a man of great experience, that the prisoner was insane."* Upon the receipt of this information, the Home Secretary directed the Medical Inspector of Prisons to visit Clark, and report upon his mental condition. This gentleman certified, in the most decided terms, to the insanity of the prisoner, and the Home Secretary respited the execution of the sentence, and wrote to the visiting justices to request that they would take the necessary steps for having the prisoner committed to an asylum. The requisite medical certificate for this purpose, signed by two physicians, who stated their opinion of the insanity of the prisoner in explicit terms, was transmitted to the Home Secretary; but the *visiting justices declined to concur in the opinion;* consequently, the requirements of the law were not satisfied which would authorize the Secretary of State to direct the removal of the prisoner to an asylum. Hence, it had become necessary for the Home Secretary to direct the attention of the visiting justices to the duty which has devolved upon them, of taking care that the prisoner is placed in such a position that he can do no injury to himself or others. Here, for the present, the question rests.

It needed but the refusal of the visiting justices to assent to a conclusion which had, after the trial, dawned upon the mind of the judge, and which had been accepted by the Home Secretary, to bring to a fitting termination this strange judicial miscarriage. We can well imagine that the visiting justices have been, and that very naturally, influenced in their decision by the opinions so emphatically expressed by the learned judge on the sanity of the prisoner in passing sentence. The evidence upon which they were called to certify the prisoner's insanity could, in no respect, be more important either in degree or significance, if, indeed, it was not in every respect the same, so far as the actual *facts* of the evidence were concerned, than that which Mr. Justice Willes had, in pronouncing sentence, so absolutely set aside as of no moment in influencing the result of the trial. It is not for us to explain in what manner these facts could avail so little to save the judgment-seat from what seems something like a mockery of justice, by calling into exercise those powers which the judge amply possessed for the postponement either of the trial or the sentence, and yet be of sufficient import to induce to take immediate steps for instituting an inquiry into the rightful ness of the sentence he had just passed being carried into effect.

The questions which arise out of the conduct of this case, the summing up, and the opinions expressed by the learned judge, whilst passing sentence, are of unusual importance; and they assume a peculiar gravity at the present moment in consequence of the opinions so recently expressed on the value of medical evidence in cases of insanity before courts of law, by the Lord Chancellor, in the discussions which arose in the House of Lords on his Lunacy Regulation Bill. On the second reading of the Bill he said :—" The introduction of medical opinions and medical theories into this subject has proceeded upon the vicious principle of *considering insanity as a disease;* whereas the law regards it as a *fact* which can be ascertained by the evidence in like manner as any other fact. Therefore, we impanel a jury of ordinary men, and call upon them to try the question by proof of the habits, the demeanour, the conversation, and the acts of the alleged lunatic."* The Lord Chancellor's remarks were directed to the question of lunacy in civil cases; but we presume the principles upon which they were based are, if right, equally applicable to criminal cases.

It is as an illustration of the assumed competence of judges and juries, unaided by medical evidence, to deal with lunacy as a simple matter of *fact,* in the signification which the Lord Chancellor appears to attach to that word, that the more permanent interest of the case of George Clark consists. Never, probably, was a case submitted to a court of justice in which the "facts" of the case were so entirely freed from extraneous or irrelevant matter, and as Mr. Justice Willes pointed out to the jury—" In one respect it was an advantage that the prisoner declined to be defended by counsel; and they had an opportunity of observing for a long time his conduct at, of course, a very critical moment of his life."

Now, what were the " facts" deposed to by the witnesses, or to be noted from the actions and language of the prisoner himself, which would enable a jury to form a just notion of his probable mental condition at the period of the act for which he was accused; and whether at that time he was, from insanity, irresponsible for his act, according to the limitations of responsibility laid down by the law?

1. And first, the language of the prisoner while being tried was characterized by singular irrelevance and want of sequence, and at times by manifest incoherence.

2. There was an entire absence of any evidence to show—nay, it was even barely suggested incidentally—that these peculiarities

* *Times,* March 12.

might be assumed; but it was deposed by different and independent witnesses that (*a*) they had distinguished the prisoner's conversation at all times several years previous to the murder; (*b*) that they were manifested also at the time of the coroner's inquest and during the examination before the magistrates; and (*c*) that they had been observed during the whole period of his imprisonment, nearly five months. That, in fact, these peculiarities had been the ordinary characteristics of the prisoner's conversation prior to, at the time of, and subsequent to the murder.

3. It was also deposed that shortly after the distraint made for the dog-tax, and about four months before the murder, the prisoner had met the distraining agent, and requested him to tell Mr. Frater that "*he had a mission to perform between God and himself, and that if the goods were not returned he* (Mr. Frater) *should pay dearly for it;*" and, if the prisoner's own statement to the jury is to be believed, he was probably subject to delusions in 1860.

4. It was further deposed that the prisoner had suffered from several *delusions* of a religious character since the murder,—to wit, (*a*) that he was Christ; (*b*) that sixty years ago (he being, according to his own statement, at the time of speaking, but 45 years of age) he had dwelt in Palestine, and *that he was sent here as a supreme power*.

5. Finally, the prisoner was apparently under the influence of delusions as to the Queen even during the time of his trial.

These were "facts" sworn to by the witnesses for the prosecution, or open to the observation of the court and jury; yet, with these facts before him, we find the learned judge remarking to the prisoner whilst passing sentence—a prisoner at the very moment clearly under the influence of delusions, and who from time to time had been incoherent,—"If I had entertained the *slightest doubt* on the evidence that you were not in a condition to understand perfectly the whole of what you were doing in depriving yourself of counsel and defending yourself, I should in the first instance have postponed the trial, and I should have postponed passing on you the sentence of the law; *but I am convinced that you are a person of eccentric conduct and violent character —your character is eccentric beyond that of other men.* No doubt you knew you were committing murder when you took Frater's life. I must, therefore, proceed to pass sentence at once, hoping that it is possible, after those declarations you have made—after the determination to take away life—*after that dreadful state of mind you have been in since, rejoicing in the crime you have committed, rejoicing in shedding the blood of a fellow-creature*— I do hope that you have sincere feelings of repentance to God, and that you will devote yourself to asking forgiveness of the Almighty, for the sake of Him who died for us all."

Did no suspicions strike the judge that there was a strange, if not revolting, inconsistency in addressing this last sentence to a man who but a few days before, and probably at that very moment, believed himself to be the, or at least a, Christ? Did no doubt whisper to his lordship that in inferring mere eccentricity from delusion and incoherence, he might be setting all trustworthy experience aside, and heralding the awful sentence which was about to pass his lips with an assumption to which the wildest theory ever advocated by a medical witness in a court of justice was the mere folly of a child? Had it never crossed the minds of the judge and the jury that there might probably be some intimate and all-important relation between the prisoner's rejoicing in the crime he had committed, his religious delusions, his message to Frater that he had a certain mission to perform between God and himself, the threat with which the message closed, and the murder?

From the prisoner's own statement to the jury, it appeared that he associated the dog-tax with certain assumed hardships of the working classes; and that the tax, although levied in the Queen's name, was disavowed by her, personally to himself, and that it was enforced for the benefit of local officials only. His was not a personal grievance, but in his person was made evident certain sufferings under which a whole class groaned. These notions of the prisoner it is most probable from the evidence adduced at the trial, and from the prisoner's own statements, existed prior to the murder of Mr. Frater; and it is very noteworthy that Mr. Gilvray had deposed that immediately after the murder the prisoner "had talked about taxes a good deal, and about paying them to the Queen, and not to any other person. He seemed to talk as if there were a fictitious Queen, and they did not pay taxes to the real one." Here the prisoner interrupting, said, "I did not say the Queens. I said the Queen was not receiving the dog-taxes, but the government was receiving them. But that will come after."

Surely it was essential to the ends of justice to know in what manner the prisoner's ideas about the dog-tax were influenced by his religious delusions: whether as Christ, or "a supreme power," he had been commissioned to experience and redress the wrongs of his fellow-workers; whether, as his words spoken four months before the murder would seem to imply, the destruction of Mr. Frater was an incidental part of his scheme of redress; whether his rejoicing at the success of his crime, was not the rejoicing of a lunatic happy in the successful accomplishment of a supposed heavenly mission, and whether his refusal to be aided by counsel, and his wild defence at the bar, were not based upon some peculiar religious fancy of a similar kind, and probably connected with, that which had induced him to drink a pint of olive oil weekly, "so that he might keep his lamp well trimmed and burning to show him to another world."

The whole history of the case, indeed, renders it highly probable that the murder was committed under the influence of an insane delusion, which would have brought the case strictly within at least the theoretical limits of the criteria of irresponsibility laid down by the judges in these words :—" It must be clearly proved that, at the time of the committing of the act, the party accused was labouring under such a defect of reason, from disease of the mind, as not to know the nature and quality of the act he was doing, *or if he did know it, that he did not know he was doing what was wrong. . . . . If the accused was conscious that the act was one which he ought not to do,* and if that act was at the same time contrary to the law of the land, he is punishable." Could either a consciousness of wrong-doing, or " malice," in the legal sense of the term, be legitimately inferred from a mental condition such as we have suggested might probably have been present in Clark's case at the time of the murder ?

But both the immediate and the more remote significance of the "facts" brought before the jury alike escaped their notice, as well as that of the learned judge. How entirely those "facts" were misapprehended is sufficiently shown by the circumstance that the judge possessed the power either of postponing the case or the sentence; and by the adoption of either course a great scandal to justice would have been avoided. If it had not been for this discretional power of postponement, avowed, by the judge, we might have admitted, and rested content with, the legal justice of the verdict of guilty, on the ground that there was no evidence, as stated by his Lordship to the Secretary of State, that the prisoner was insane at the time when the crime was committed. But there was the most positive and undoubted evidence before the court, that as was the prisoner's mental condition at the time of the trial, so it had been for years previous to the crime of which he was accused, and at all periods of the intervening space ; and that he had suffered from religious delusions as well prior to as subsequent to the crime. *There was not one particle of evidence before the court that the facts indicative of the mental state of the prisoner, in the period mentioned, were otherwise than significant of mental disease.* How came it to pass, then, that the discretional power of the judge was not exercised, in order that the justice of the case might be met by a specific investigation of these facts in their bearing upon the question at issue ? We cannot for a moment suppose that the opinion expressed by the surgeon of the police force, of the prisoner's sanity at the time of the murder, could have influenced either the judge or the jury; for had this been the case, from what cause did it happen that his still more positive opinion, *supported by the statement of definite facts* and by the evidence of the gaol chaplain, of the then insane state of the prisoner, had no weight with the court, and had so little influence with the jury, that they did not even have recourse to that ready device for obviating undue harshness in the administration of the law, by appending a recommendation to mercy, on account of insanity, to their verdict? It is not less curious to note, that while the surgeon's opinion on the mental state of the lunatic at the time of the crime —the very period when of all others it was necessary to obtain accurate information, and admit no opinion except when based upon clearly ascertained facts—it is most curious to note that while an opinion relating to this period was suffered by the judge to pass unchallenged, the opinions as to the then mental condition of the prisoner were required to be supported by statements of fact!

Need we pursue our examination of this painful case further? Here we have the strange spectacle of one of the most learned and most justly honoured judges of the realm, and a jury of respectable householders, listening for several hours to the wild pleadings, incoherencies, and delusions of a lunatic suffering from one of the commonest and most familiarly known forms of lunacy; receiving from independent sources the most positive and unquestionable depositions of facts testifying to that lunacy over the period to which their inquiry referred; yet so utterly misapprehending these facts, so entirely ignoring their more patent significance, as not only to misinterpret them, but also to suffer the essential question of the responsibility or irresponsibility of the prisoner to remain unsolved—nay, even without an attempt to solve it—and unhesitatingly assuming that he was then, and had been at all times, within the cognizance of the court, a sane man! And as such the jury pronounced the prisoner guilty, and the judge sentenced him to death, without one thought of mercy—a mercy so little niggard of its blessings that it can shield the drunkard, who, in the mere wantonness of brutality, kills a honoured householder, the father of a large family, on the threshold of his own dwelling;* and save from the gallows the justly-pitied youth who, angered by injustice and ill-usage, had stabbed to death the lodging-house keeper who had so infamously victimized him.†

---

* Case of Quail, aged 22, and others, tried at the Central Criminal Court, on the 4th of March, 1862, for the murder, in a drunken freak, of John Wincott, a butcher, residing in Marylebone. A verdict of manslaughter was returned, in the teeth of the summing up of the Chief Baron, and the prisoner was subsequently sentenced, by Baron Martin, to *six years* penal servitude.

† Case of Patrick Devereux, aged 19, tried for the murder of James Gardner at the Central Criminal Court, March 5, 1862. A verdict of guilty was returned coupled with a recommendation to mercy on account of the youth of the culprit. The sentence was commuted to *penal servitude for life.*

Is it, then, by the light of this case of George Clark that we are to read the legal doctrine of the evidence of "fact" in cases of lunacy? Is this case calculated to give us confidence that the judges of the realm are competent, unaided by medical evidence, to deal with the "fact" of lunacy as a fact, with a due regard not merely to the requirements of justice, but even of the strict letter of the law of the land? Is the exexistence of insanity as a fact, inconsistent with the fact of insanity as a disease? Is it possible to entertain a conception of insanity as a fact without at the same time conceiving it to be a disease? Is not, indeed, the conception of insanity as a disease essential even to the legal notion of insanity as a fact? Is not this the necessary conclusion to be attached to the statement of the judges, that, in criminal cases, "to establish a defence on the ground of insanity, it must be clearly proved, that, at the time of the committing of the act, the party accused was labouring under such a defect of reason *from disease of the mind*," as they set forth? Is a different signification attached to the fact of insanity in civil law, to that which is attached to it in criminal? Is not the notion of disease the essential element in the conception of insanity as a fact? Can a rule of law make any fact *absolute*—that is, otherwise than what it is? "All facts involve ideas," says the distinguished author of the *Novum Organon Renovatum*. "Since then in observing facts, we cannot exclude ideas, we must, for the purposes of science, take care that the ideas are clear and vigorously applied" (Aph. iv.) Can the law stand aloof from this great truth? Does not this truth, indeed, underlie of necessity the legal doctrine of fact? What was the *idea* which entered into Mr. Justice Willes' conception of the "fact" of insanity, and into the conceptions of the jurymen at Newcastle? Would not this idea unavoidably be based either on personal observation of cases of insanity or on the recorded experience of other observers? Would not the value of both the judge's and the jury's opinion as to the "fact" of insanity be in direct proportion to the actual experience or the theoretical knowledge of insanity which had fallen to the lot either of the one or the other? Is it not the assumed greater experience of the medical man in studying insanity as a question of "fact," which gives weight to his opinion in the witness box? Where, then, is the great legal difficulty of dealing with medical evidence? Is the fact of experience to be assumed in any case without direct proof of its existence? and being proved, does the experience carry the least weight apart from the "facts" of any case sought to be cleared by the aid of that experience? Is not, in fact, the medical man had recourse to, in the witness box, solely to aid the court or the jury, or both, in their interpretation of certain asserted "facts?" Must not his opinion fall to the ground or stand with the substantiation or not of these "facts?" And is it not the peculiar function of the jury to determine the value of the asserted facts? Without medical evidence "facts" of the utmost significance, and the true interpretation of which is essential to the ends of justice, may, as in the case of George Clark, be entirely misapprehended, to the great scandal of the administration of the law; but the medical evidence is of value only so far as the "facts" upon which it is based can be made evident to the jury. Many of these "facts" the medical man can only assume; he cannot submit them to a legal test, but can judge of them only by those rules of evidence which govern men in ordinary life. Here it is that the great source of difference of medical evidence arises —a difference, as a rule, more apparent than real; and it is disingenuous of lawyers to contemn medical evidence on account of this discrepancy, seeing that it is a necessary result of legal pleadings, and that it is fostered by the very nature of these pleadings. If this be true, it is to the tact of the judge, and not to arbitrary rules of law, that we must look for any satisfactory relief from the obnoxious phases of medical evidence.

To depreciate medical evidence in cases of lunacy is to seek to set aside whatever light has been thrown in the past half-century upon this complex affection; it is to subject to popular apprehension questions in the decision of which even the most acute minds might well hesitate; it is to sacrifice the interest of the individual to the prejudice of the many. Insanity is a fact not limited by its legal bearings; it has a much wider scope and higher interest. It is the fruitful source of untold misery, wretchedness, and pain, escape from which is alone possible by the recognition of the truth that insanity is a *disease*, amenable, as other diseases, to the care and treatment of the physician. Except as a disease, the very notion of insanity falls to the ground, and the existence of this disease is inferred upon the same principles of observation and reasoning as the existence of any other disease. Insanity, indeed, is a fact of inference, not a fact *per se*—a fact deduced from other facts, not a primary fact; and this deduction is not one of so glaring a character that it is patent to every one, as the case of George Clark most conclusively and instructively shows.

How it could come to pass that the prisoner, a manifest lunatic in the opinions of at least two of the gaol officials, was suffered by the police authorities of Newcastle to plead to his accusation, without due representations, supported by the requisite medical evidence, having been previously submitted to the presiding judge, is not the least singular phase of this extraordinary case.

